# Insight into the Role of the PI3K/Akt Pathway in Ischemic Injury and Post-Infarct Left Ventricular Remodeling in Normal and Diabetic Heart

**DOI:** 10.3390/cells11091553

**Published:** 2022-05-05

**Authors:** Bartosz Walkowski, Marcin Kleibert, Miłosz Majka, Małgorzata Wojciechowska

**Affiliations:** 1 Laboratory of Centre for Preclinical Research, Department of Experimental and Clinical Physiology, Medical University of Warsaw, Banacha 1b, 02-097 Warsaw, Poland; walkowski.bartosz@wp.pl (B.W.); malgorzata.wojciechowska2@wum.edu.pl (M.W.); 2 Invasive Cardiology Unit, Independent Public Specialist Western Hospital John Paul II, Daleka 11, 05-825 Grodzisk Mazowiecki, Poland

**Keywords:** PI3K/Akt pathway, myocardial infarction, heart failure, left ventricular remodeling, diabetes, apoptosis, necroptosis, ferroptosis, pyroptosis, fibrosis

## Abstract

Despite the significant decline in mortality, cardiovascular diseases are still the leading cause of death worldwide. Among them, myocardial infarction (MI) seems to be the most important. A further decline in the death rate may be achieved by the introduction of molecularly targeted drugs. It seems that the components of the PI3K/Akt signaling pathway are good candidates for this. The PI3K/Akt pathway plays a key role in the regulation of the growth and survival of cells, such as cardiomyocytes. In addition, it has been shown that the activation of the PI3K/Akt pathway results in the alleviation of the negative post-infarct changes in the myocardium and is impaired in the state of diabetes. In this article, the role of this pathway was described in each step of ischemia and subsequent left ventricular remodeling. In addition, we point out the most promising substances which need more investigation before introduction into clinical practice. Moreover, we present the impact of diabetes and widely used cardiac and antidiabetic drugs on the PI3K/Akt pathway and discuss the molecular mechanism of its effects on myocardial ischemia and left ventricular remodeling.

## 1. Introduction

Cardiovascular diseases (CVD) are the leading cause of death globally, and myocardial infarction (MI) is of fundamental importance [1]. MI is caused by the restriction of blood flow through the coronary arteries, which results in an imbalance between myocardial demand and the blood supply. It results in the activation of many intracellular pathways which promotes the activation of programmed and unprogrammed cell death, leading to the impairment of cardiac functions [2,3]. The most common cause of MI is a rupture of an atherosclerotic plaque. Numerous risk factors such as smoking, a high level of low-density cholesterol, hypertension, diabetes mellitus, and a lack of physical activity stimulate atherosclerotic plaque progression. The complexity of atherosclerosis progression and vulnerability of plaques were well-described by Libby et al. and Anderson et al. [4,5].

The 1-year mortality rate among patients after MI is still high [6]. However, improvements in the acute treatment of MI have increased the number of surviving patients, who are at higher risk of recurrent infarction and development of post-infarction heart failure (HF) [7,8,9]. 

At the same time, cardiovascular diseases are associated with growing public and private expenditure on healthcare worldwide [10]. Therefore, a new therapeutic approach is needed to reduce the cost of CVD treatment. It seems that targeted modification of molecular pathways may improve the outcome for patients.

Post-infarction HF is mostly caused by adverse remodeling of the left ventricle (LV). This is a series of molecular, cellular, and interstitial changes following MI [11,12,13]. It involves the inflammation, fibrosis, and hypertrophy of the myocardium, and changes in the vascular bed and the conducive system of the heart [14,15]. Altered remodeling results in electrophysiological disorders and ventricular dysfunction and leads to heart failure, which significantly impacts a patient’s prognosis [16,17,18,19]. One of the main risk factors of post-MI HF development is diabetes mellitus (DM), which impairs cell communication, involving many molecular pathways including PI3K/Akt [20]. 

The phosphatidylinositol 3-kinase (PI3K)/protein kinase B(Akt) pathway is one of the most significant intracellular signal transduction pathways. Even though it was relatively recently discovered, it has caused a revolution mainly in personalized oncology. PI3K was identified by Lewis Cantley’s group in 1988 [21], while Akt kinase was identified by Stephen Staal in 1987 [22]. It has become a subject of great interest in the scientific community and the focus of numerous basic and clinical studies. 

The proper activity of PI3K and Akt appears to be essential for the development, functioning, and survival of the organism. The lack of their expression leads to a blockade of cell division and embryonic lethality [23,24]. In response to extracellular signals, the PI3K/Akt pathway controls cell metabolism, growth, proliferation, and the stress response. It plays a key role in the proper functioning of most human organs and is associated with the development of diseases [25,26,27]. In the myocardium, Akt is a central signal transductor, and the PI3K/Akt pathway is responsible for proper metabolism and cell response. Indeed, it plays a role in cardiovascular diseases, including chronic (CCS) and acute coronary syndromes (ACS) or HF. In this review, we summarize the currently known role of the PI3K/Akt pathway in the pathophysiology of myocardial infarction and the post-infarction remodeling of the left ventricle, and we indicate the potential targets of therapy. In addition, we discuss the clinical importance of the impact of diabetes and commonly used drugs on its activity in the heart.

## 2. The PI3K/Akt Pathway and Its Impact on the Peri-Infarct Processes

**Figure 1:**
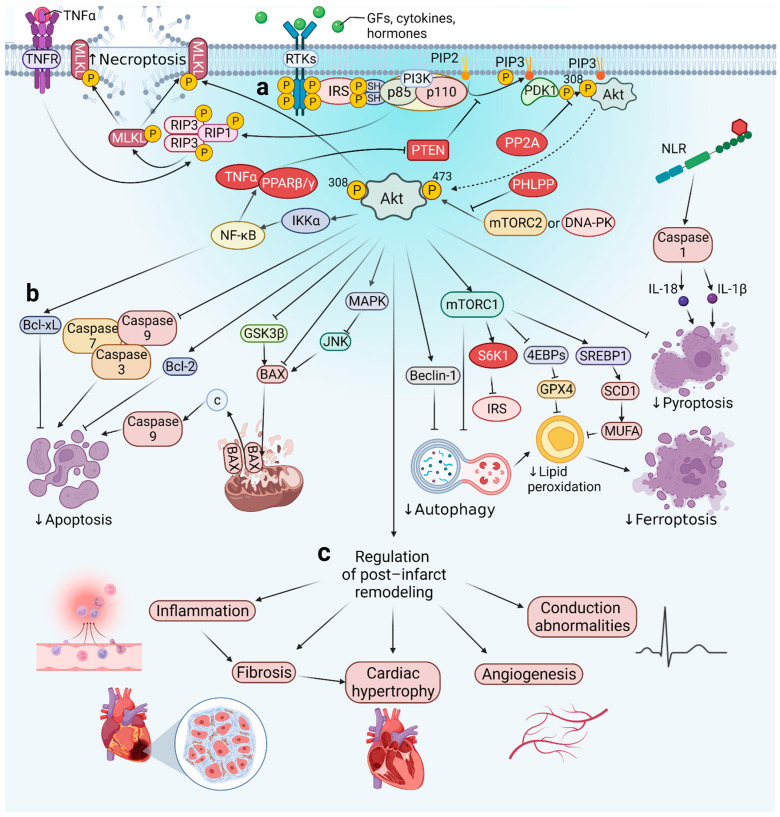
Schematic of components and mechanisms of PI3K/Akt activation and regulation (**a**). PI3K/Akt pathway regulates cell death during MI (**b**). PI3K/Akt signaling is a key component in the regulation of numerous processes in the peri-infarct period and post-infarction remodeling of the left ventricle (**c**). Akt—protein kinase B, Bax—Bcl-2-associated X protein, Bcl-xL—B-cell lymphoma-extra large, Bcl-2—B-cell lymphoma 2, c—cytochrome c, DNA-PK—DNA-dependent protein kinase, GFs—growth factors, GPX4—glutathione peroxidase 4, GSK3β—glycogen synthase kinase 3 β, IKKα—IκB kinase α, IL-1β, IL-18—interleukins 1β and 18, IRS—insulin receptor substrate, JNK—c-Jun NH_2_-terminal kinase, MAPK—mitogen-activated protein kinase, MUFA—monounsaturated fatty acids, MLKL—mixed lineage kinase domain-like protein, mTORC1, mTORC2—mammalian target of rapamycin complex 1 and 2, NF-κB—nuclear factor κ-light-chain-enhancer of activated B cells, NLR—NOD-like receptor, P—phosphate, PDK1—phosphoinositide-dependent kinase-1, PHLPP—pleckstrin homology domain leucine-rich repeat protein phosphatase, PIP2—phosphatidylinositol-4,5-bisphosphate, PIP3—phosphatidylinositol-3,4,5-trisphosphate, PI3K—phosphoinositide 3-kinase, PPARβ/ɣ—peroxisome proliferator-activated receptor β/ɣ, PP2A—protein phosphatase 2, PTEN—phosphatase and tensin homolog, RIP1, RIP3—receptor-interacting protein 1 and 3, RTK—receptor tyrosine kinase, SCD1—stearoyl-CoA desaturase-1, SREBP1—sterol regulatory element-binding protein-1, S6K1—ribosomal S6 kinase-1, TNFα—tumor necrosis factor α, TNFR—tumor necrosis factor receptor, 4EBPs—eukaryotic translation initiation factor 4E-binding protein 1.

The main molecules involved in the PI3K/Akt pathway are presented in the figure below (Figure 1a) This pathway consists of tyrosine kinase receptors (RTKs), phosphatidylinositol 3-kinase (PI3K), phosphatidylinositol-4,5-bisphosphate (PIP2), phosphatidylinositol-3,4,5-bisphosphate (PIP3), and Akt (Figure 1a).

### 2.1. Components and Mechanisms of PI3K/Akt Activation

Receptor tyrosine kinases (RTKs) are surface receptors composed of three functional domains: an extracellular ligand-binding domain, a transmembrane domain and an intracellular tyrosine kinase domain [28]. After bounding a ligand (growth factors (GFs), cytokines, hormones), RTK forms a dimer, which activates the intracellular domain and the mutual autophosphorylation of each monomer [29]. PI3K kinase consists of two domains: catalytic P110 and regulatory P85, which can be activated by RTK or indirectly by adapter molecules (including the insulin receptor substrate IRS, or the GTP-binding protein RAS) [29,30]. Activated PI3K phosphorylates the hydroxyl group in the third position of the inositol ring of phosphatidylinositol [31]. Phosphatidylinositol-4,5-bisphosphate (PIP2) and phosphatidylinositol-3,4,5-trisphosphate (PIP3) are small phospholipid components of cell membranes and important signaling transductors. In the PI3K/Akt pathway, the 3-position PIP3 phosphate group can bind to both phosphoinositide-dependent kinase-1 (PDK1) and Akt (also known as PKB (protein kinase B)) and recruit the Akt protein in the cell membrane, enabling PDK1 to access the PKB [32]. Akt/PKB is a highly conserved serine/threonine kinase. It plays a key role in many cellular processes and serves a dominant role in the signal transduction of the entire PI3K pathway. The amino acid structure of Akt, from the N-terminus to the C-terminus, consists of three recognizable domains: a pleckstrin homology (PH) domain, the central catalytic domain, and the carboxy-terminal regulatory domain. The PH domain acts as a mediator in membrane translocation following Akt activation, whereas the catalytic domain binds ATP. Complete activation of Akt requires phosphorylation by PDK1 at the T308 site [33] and by the mammalian target of rapamycin complex 2 (mTORC2) [34,35] or DNA-dependent protein kinase (DNA-PK) [36] at the S473 site. Activated Akt is transported from the cell membrane to other regions of the cell to phosphorylate its substrates, and consequently, either to suppress or enhance their activity. Therefore, Akt mediates diverse important cellular processes, including cell growth and proliferation, cell survival, and gene expression, as detailed in later sections of this article [37]. Its three isoforms are distinguished, namely Akt1, Akt2, and Akt3 (also known as PKBa, PKBb, and PKBc, respectively) [38,39]. Their expression varies between tissues and disease states [40,41]. 

### 2.2. Regulation of the PI3K/Akt Signaling

Due to the importance of this pathway, its activity must be carefully regulated. Downregulation of the PI3K/Akt pathway may be achieved via two mechanisms. Firstly, phosphatase and tensin homolog (PTEN) may specifically dephosphorylate the 3-phosphate of the inositol ring in PIP3 to PIP2 and therefore reduce the concentration of PIP3 required for PI3K activation [42,43,44]. Inactivation of PTEN results in the constitutive activation of Akt and the mammalian target of rapamycin complex 1 (mTORC1), which is an evolutionarily conserved regulator of translation and ribosome biogenesis. Therefore, it leads to the disturbance of the size of the cells and growth regulation [45]. Another mechanism is dephosphorylation of Akt either at Thr308 by protein phosphatase 2A (PP2A) or at Ser473 by pleckstrin homology domain leucine-rich repeat protein phosphatase (PHLPP) [46]. The PI3K/Akt pathway itself also has feedback mechanisms. Akt phosphorylates IκB kinase α (IKKα) at the Thr23 site, which in turn phosphorylates nuclear factor κ-light-chain-enhancer of activated B cells (NF-κB). Activation of NF-κB regulates peroxisome proliferator-activated receptor delta (PPARβ/δ) agonists and tumor necrosis factor α (TNFα), which in turn repress PTEN expression as positive feedback. In addition, a negative feedback loop is initiated by mTORC1 and ribosomal S6 kinase-1 (S6K1) activation, which phosphorylates the insulin receptor substrate (IRS-1) and prevents its binding to RTKs, which results in the suppression of PI3K.

### 2.3. Impact of PI3K/Akt on Peri-Infarct Processes

The activity of the described pathway has an impact on many processes associated with the peri-infarct period, such as myocardial fibrosis, and other components of post-infarct left ventricular remodeling (Figure 1b,c). All of them will be described one-by-one in the context of PI3K/Akt. In addition, the influence of some comorbidities and commonly administered drugs in all of these processes is discussed. 

## 3. Activity of the PI3K/Akt Pathway in Myocardial Infarction

Both the physical obstruction of a coronary vessel and the redistribution of blood flow reduce the oxygen supply and are responsible for irreversible damage of the cardiomyocytes [47]. Necrosis has been recognized as the main pathway of cardiomyocyte death during MI. However, the role of programmed types of cell death in ischemic and post-infarct heart injury has gained attention over the past two decades. Their activity determines the size of the infarcted area, and consequently, impairs the functions of the myocardium [48]. Inhibition of these processes, both pharmacologically and genetically, could improve cardiac functions. Moreover, the recently common division of cell death into programmed apoptosis and unregulated necrosis is slowly being turned into the field of study of MI. Recent studies also indicate necroptosis, ferroptosis, pyroptosis, and parthanatos as programmed types of cell death, also involved in MI pathogenesis [49,50,51]. All these processes ultimately result in the loss of the integrity of the cell membrane and the inflammatory response triggered by the release of the cytoplasmic content into the environment. The PI3K/Akt pathway appears to be directly involved in many of these processes (Figure 1b). For this reason, its proper regulation may contribute to reducing the damage caused by MI. The sections below describe these impacts in detail.

### 3.1. Necrosis

Necrosis is the unprogrammed type of cell death that is responsible for infarct size to the greatest extent. The beginning of necrosis is the imbalance between myocardial demand for oxygen and blood supply during MI, which causes the activation of anaerobic metabolism. This leads to the intracellular accumulation of H^+^, which arises from anaerobic glycolysis. In addition, the lack of ATP causes the dysfunction of the Na^+^/K^+^ ion pump. The increased level of H^+^ causes the need for their removal by the Na^+^/H^+^ exchanger. In response to elevated levels of Na^+^ (associated with the Na^+^/K^+^ ion pump failure and the Na^+^/H^+^ exchanger) inside of the cell, the Na^+^/Ca^2+^ exchanger operates in reverse mode and causes an increase in Ca^2+^ concentration. Elevated levels of all of these ions cause the swelling of the cell and promote the rupture of the cell membrane and necrosis. In one basic study, it was shown that pretreatment with HDL can protect the cardiomyocytes against oxygen and glucose deprivation-dependant necrosis. In addition, the authors brought about the inhibition of the PI3K/Akt pathway which abolished the protective effect of HDL [52]. Moreover, the PI3K/Akt pathway is associated with the generation of reactive oxygen species (ROS) which enhance necrosis [53,54,55]. There is evidence from numerous studies on cancer cells that disturbed signaling within this pathway contributes to increased levels of ROS. This may occur both directly through the modulation of mitochondrial bioenergetics and the activation of NADPH oxidases (NOXs), and indirectly, where ROS is produced as a metabolic by-product [54,56,57,58,59]. Necrosis is involved in most of the myocardial post-infarct changes. Due to cell membrane rapture, it activates and promotes inflammation in the ischemic heart, which is the inducer of other processes such as fibrosis, hypertrophy, and hemodynamic dysfunction, which are responsible for the adverse remodeling of the left ventricle. 

### 3.2. Programmed Type of Cell Death

#### 3.2.1. Apoptosis

Cardiomyocyte apoptosis can be induced by the intrinsic pathway in response to DNA damage, increased ROS and cytosolic calcium levels, or by the extrinsic pathway as a consequence of activation of sarcolemmal death receptors (FAS or TNFα) [60]. During MI, the PI3K/Akt pathway activity downregulates the expression of numerous proapoptotic molecules, which are briefly described in this section [61,62,63]. Their increased expression is associated with a higher activity of apoptosis and an increase in the number of cells dying as a result of this type of cell death. For instance, Akt phosphorylates cysteine-aspartic proteases (caspases) such as caspase-3, caspase-7, and caspase-9 (at Ser196). This prevents a caspase cascade, leading to cell death [64]. 

Moreover, the PI3K/Akt pathway upregulates the expression of the anti-apoptotic molecule B-cell lymphoma 2 (BCL-2), which blocks the process of the formation of the mitochondrial pore by which cytochrome c is released. Consequently, once PI3K/Akt signaling is active, the release of cytochrome c, which induces apoptosis by the intrinsic pathway, is reduced [65,66]. Moreover, Kim et al. showed in a cell culture that Akt kinase can decrease the activity of apoptosis signal-regulating kinase 1 (ASK1) [67]. ASK1 is a mitogen-activated protein kinase (MAPK) and can be activated by multiple cytotoxic stressors [68]. Akt phosphorylates ASK1 at Ser83 and thus inhibits it. This consequently suppresses the activity of c-Jun N-terminal kinase (JNK) and activating transcription factor 2 (ATF-2) in intact cells, which finally results in the inhibition of apoptosis induced by ASK1 [67].

In addition to the abovementioned, Akt also phosphorylates molecules such as BCL-2 associated agonist of cell death (BAD) and glycogen synthase kinase 3 beta (GSK3β), which results in the decreased activity of apoptotic pathways [69]. Blume-Jenes et al. [70] revealed in cell line studies that PI3K/Akt can also regulate cell death of its downstream factors. For example, it phosphorylates Ser136 residues of the BAD protein, a pro-apoptotic protein of the BCL-2 family, thereby depriving the apoptotic complex of its function. This causes translocation from the mitochondrial membrane to the cytosol and consequently negatively regulates the pro-apoptotic activity of Bax. Interestingly, Akt can also phosphorylate BAD at other sites, such as Ser99, Ser75, and Ser118 [71,72,73]. Phosphorylation of GSK3β on a highly conserved N-terminal regulatory site at Ser9 contributes to both myocardial necrosis (by mitochondrial permeability transition pore (mPTP) opening) and apoptosis (via various mechanisms including phosphorylation of Bax and destabilization of pro-survival beta-catenin) [74,75]. Bax can also be phosphorylated at residue S184 directly by Akt, which inhibits its conformational change for mitochondrial membrane distribution [76,77]. 

Statins can reduce apoptosis through the PI3K/Akt pathway. It was shown in animals that pitavastatin encapsulated in nanoparticles can activate this pathway and significantly reduce the TUNEL-positive cells in comparison with pitavastatin alone and a placebo. The protective effect of this lipid-lowering drug was diminished by a PI3K/Akt pathway inhibitor (wortmannin) [78]. In addition, it was shown that this pathway can be important in some pleiotropic effects of statins, such as improvement of endothelial function (defined as increased NO production), increases in proliferation and migration, and reduction in apoptosis of cardiac microvascular endothelial cells [79]. Additionally, angiotensin-converting enzyme inhibitors (ACEIs) may have a positive impact on cardiomyocytes. One of the ACEIs (benazepril) reduced apoptosis activity in an in vitro model of doxorubicin cardiotoxicity (H9c2 cell line). This positive effect was mediated by the activation of the PI3K/Akt pathway. It was shown that benazepril treatment restored the phosphorylation of Akt reduced by the doxorubicin therapy. In addition, the administration of an Akt inhibitor diminished the cardioprotective effect of this ACEI. [80]. Due to the fact that ACEIs are commonly used drugs among all patients after MI, an experiment which assesses the role of this group of drugs in apoptosis regulation in the context of the PI3K/Akt pathway is needed. The reduction in apoptosis activity and the improvement of the hemodynamic function after MI was also reported as an effect of eplerenone and bisoprolol [81,82]. Furthermore, the combination of drugs may have an even better impact on cardiomyocyte survival than therapy with a single substance. It was shown in animals that a combination of rosuvastatin with carvedilol significantly reduced the increase in biomarkers of MI (such as troponin and CK-MB). The authors observed the simultaneous activation of the PI3K/Akt pro-survival pathway [83].

Moreover, Liu et al. revealed that PI3K/Akt/nuclear factor signaling erythroid 2-related factor 2 (Nrf2), by upregulating the expression of heme oxygenase-1 (HO-1), can protect H9c2 cardiomyocytes from ischemia-reperfusion injury (IRI)-induced apoptosis [84]. They administered hydroxysafflor yellow A (HSYA), a chemical compound with a previously noticed protective effect [85,86], during reperfusion. This resulted in the increased expression and activity of HO-1, Akt phosphorylation, translocation of nuclear factor Nrf2, and, consequently, a decrease in apoptosis. Inhibition of PI3K by LY294002 abolished these positive effects.

In addition, Feng et al. showed in their study on mice that ischemia induces the expression of PTEN and the levels of this PI3K/Akt inhibitor remain elevated in the post-infarct period [65]. They selectively inhibited PTEN with bisperoxovanadium 5-hydroxipyridine-2-carboxylic acid (BPV(HOpic)) (1 mg/kg) in mice with MI induced by the ligation of the left anterior descending artery (LAD), and observed that BPV treatment promoted angiogenesis and reduced cardiomyocyte apoptosis, resulting in reduced infarct size. Taken together, such improved cardiac function is a result of PI3K/Akt pathway activation, which leads to a reduction in the number of cardiomyocytes undergoing apoptosis in the infarcted tissue compared with the myocardium where this pathway is inhibited [61,62,65]. Additionally, Wang et al. showed on a swine model that both mRNA and the protein level of PTEN increase after ischemia. This process was attenuated by atorvastatin treatment, which confirms that this type of lipid-lowering drug can have a positive impact on the survival of cardiomyocytes [87].

#### 3.2.2. Necroptosis

Necroptosis is associated with both physiological and pathological processes, including embryonal development, inflammatory reaction, and IRI [50,88]. In addition, it is one of the key pathways in the loss of functional cardiomyocytes during MI [89,90]. The main stimulus of necroptosis is TNFα, which once combined with its receptor (TNFR) leads to the activation of the pathway consisting of receptor-interacting serine/threonine-protein 1 (RIP1) and RIP3 and the mixed kinase domain-like protein (MLKL) pathway [91,92]. PI3K/Akt signaling appears to have a key role in promoting this process by phosphorylating and oligomerizing RIP1, RIP3, and MLKL in response to TNFα stimulation [93,94]. Inhibition of both PI3K (its catalytic subunit p110α) and Akt in mouse fibrosarcoma L929 led to the inhibition of necroptosis by the suppression of the above mediators [95].

Tuuminen et al. (2016) showed that simvastatin may affect the necroptosis activity. Pretreatment of both rat donors and recipients of the transplanted heart with simvastatin can reduce RIP kinase-1 and kinase-3 activity in comparison with the placebo. In addition, it reduced expression of caspase-3 and caspase-9 involved in apoptosis [96]. Therefore, inhibition of PI3K may prevent necroptosis and increase cell viability and heart function in MI patients.

#### 3.2.3. Ferroptosis

Ferroptosis is a relatively recently discovered iron-dependent regulated type of cell death with growing importance in MI pathogenesis, especially in its early and middle stages [97,98,99,100]. The PI3K/Akt pathway has been shown to play a significant role in its regulation [101]. 

For instance, Sun et al. [102] demonstrated in a cardiomyocyte (H9c2) cell culture that PI3K/Akt activation has a cardioprotective effect by reducing oxidative stress and inhibiting ferroptosis induced by doxorubicin (DOX) and lapatinib (LAP) [103,104]. These substances inhibit both PI3K and Akt phosphorylation and alter the mitochondrial membrane potential, reduce ATP, and increase the level of cytochrome C. Activation of the PI3K/Akt pathway using 30 μM 740Y-P resulted in a reversal of the deleterious effects of DOX and LAP and increased survival of the H9c2 cardiomyocytes [105,106]. Moreover, Yi et al. indicated that PI3K/Akt signaling inhibits ferroptosis in cancer cells via a pathway consisting of mTOR complex 1 (mTORC1), sterol regulatory element-binding proteins 1 (SREBP1) and stearoyl-coenzyme A desaturase 1 (SCD1), where SCD1 finally leads to the production of monounsaturated fatty acids [107]. Interestingly, they revealed that lipogenesis appeared to protect cells from oxidative stress and ferroptotic death.

Studies on the impact of the PI3K/Akt pathway on ferroptosis and cardiomyocyte viability are limited [107,108]. Recently, Jiang et al. published results of a bioinformatic analysis that revealed that the expression of 17 ferroptosis-related genes can be associated with the presence and development of ischemic and idiopathic cardiomyopathy. These genes were mainly involved in the regulation of apoptosis, cellular response to FGF stimulus, and response to some unspecified drugs by the MAPK and PI3K/Akt pathways. Moreover, the authors proposed some drugs and substances which can potentially be used in treatment [109]. This study provides useful information for further preclinical studies evaluating the role of PI3K/Akt signaling in the regulation of ferroptosis during MI and post-infarction HF. 

#### 3.2.4. Pyroptosis

Pyroptosis is a death pathway that begins with the activation of one of the NOD-like receptors (NLRs), which then leads to the activation of caspase-1 that finally activates pro-inflammatory cytokines, including interleukin-1β (IL-1β) and interleukin-18 (IL-18) [110]. As levels of these molecules have been found to increase during MI, inducing inflammation in the myocardium, regulation of this pathway may result in a degree of damage to the cardiomyocytes during infarction [111]. Overactivation of this highly inflammatory pathway negatively affects the infarct area and impairs myocardial contractility [112,113,114]. Although this type of cell death is most induced after infection with intracellular pathogens, its important effect has been noted during IRI.

Recently, Gio et al. [111] indicated that the radioprotective 105 kD protein (RP105)/PI3K/Akt pathway is directly involved in the regulation of pyroptosis. After the administration of piperine to rats, they observed the inhibition of miR-383, which led to the activation of RP105/PI3K/Akt signaling. A consequence of the observed increased activity of the PI3K/Akt pathway was a decrease in pyroptosis occurring in the myocardium, and thus a greater survival of cardiomyocytes [115,116,117]. 

In addition, Do Carmo et al. [113] observed that administration of VX-785, a clinically available, highly selective caspase-1 inhibitor, reduced the infarct size in rats during analysis performed on Langendorff-perfused rat hearts. Moreover, the administration of a PI3K inhibitor—wortmannin—abolished all protective effects. This is more proof of the importance of the PI3K/Akt pathway in the protection of cardiomyocytes against pyroptosis.

Although pyroptosis is a recently known and still little-understood pathway of cell death, the proven influence of PI3K/Akt in its course offers further potential therapeutic targets in alleviating MI-induced death of the cardiomyocytes. However, further investigation is needed.

### 3.3. Ischemic Conditioning

The development of a novel strategy to limit infarction is of great clinical importance because the main predictor of a patient’s prognosis is infarct size. One of the special interests is ischemic conditioning (IC)—a leading paradigm of cardioprotection [90]. IC means induction of short periods of myocardial ischemia and reperfusion before the onset of MI (preconditioning) or during reperfusion (postconditioning). Applying these cycles at a remote site is referred to as remote ischemic conditioning (RIC). In contrast to ischemic preconditioning, whose beneficial effects may be considered for patients with pre-infarction angina, ischemic postconditioning can be applied for patients undergoing PCI [90]. Apart from mechanical interventions, certain groups of drugs, such as volatile anesthetics and G protein-coupled receptor (GPCR) agonists, can also initiate pharmacological IC [118]. Moreover, exercise and pre-infarction angina can also promote transduction signals of IC, which can reduce infarct size and improve left ventricular function [119].

PI3K/Akt signaling is considered to be the main pro-survival kinase cascade mediating the IC-induced protective effect by forming a RISK pathway parallel to MEK1-ERK1/2 [90,120]. PI3K activity is required during both the pre-ischemic trigger phase and the post-ischemic mediator phase of IC to reduce infarct size and mortality [121,122]. In addition, IC increases the levels of Akt phosphorylation during both phases of IC. It plays a pivotal role as a mediator of IC and its pharmacological inhibition abolishes the infarct size either during the trigger phase or at reperfusion [123,124]. However, Barsukevich et al. showed that the role of the PI3K and RISK pathways may depend on the time of applied ischemia in the case of postconditioning. Immediate IC (10 s of reperfusion) increases phosphorylation of PI3K-AKT and ERK1/2, while early or delayed IC (applied after 10 min or 30 min of reperfusion) had no effect on phosphorylation in the rat model [125]. Nevertheless, postconditioning applied for 10 s, or 10, 30, 45, or 60 min after the onset of reperfusion retains the cardioprotective function, which is possibly dependent on a different mechanism. Another study showed that remote postconditioning increases phosphorylation of Akt and ERK1/2 in the early stages of reperfusion and then it gradually decreases [126]. Moreover, inhibition of PTEN may be important for increasing the activity of the PI3K/Akt signal in the course of postconditioning [127]. Furthermore, intermittent hypoxia increases the activity of the PI3K/Akt signal and enhances Ser473 Akt phosphorylation in the mouse model. It was associated with an increased capillary network in the myocardium and improved cardiac function, as well as reduced infarct size [128]. Administration of wortmannin (a PI3K inhibitor) reduces the level of Akt phosphorylation and its beneficial effects. This suggests that PI3K/Akt plays an important role in long-term preconditioning. An increasing number of studies indicates that extracellular vesicles (EVs) may play an essential role in mediating IC. EVs obtained from IC rats ameliorate IRI via activating the PI3K/Akt pathway. They decrease apoptosis and reduce the infarct size by increasing phosphorylation of PI3K and Akt [129]. Application of a PI3K inhibitor reverses these effects. Moreover, a study undertaken by Lessen et al. showed that EVs from the plasma of healthy human volunteers after RIC may not only provide cardioprotection via increasing mTOR expression, but also demonstrate accumulation in the damaged myocardium compared with sham-operated hearts [130]. Therefore, EVs may provide new therapeutic options for alleviating myocardial IRI via the PI3K/Akt pathway. All these observations highlight the importance of the PI3K/Akt pathway during MI and confirm its protective role.

## 4. The Role of the PI3K/Akt Pathway in Post-Infarction Left Ventricular Remodeling

**Figure 2:**
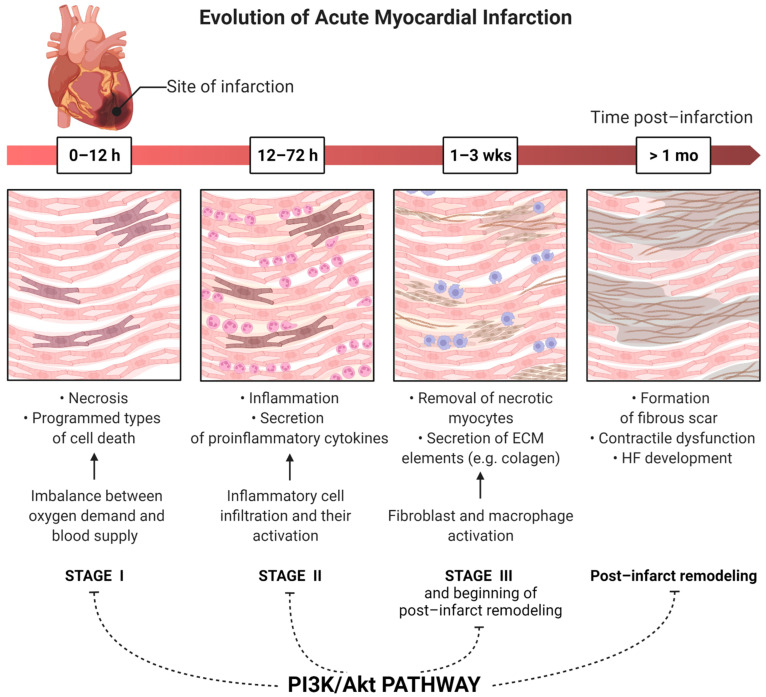
The processes regulated by the PI3K/Akt pathway which are involved in the histologic evolution of acute myocardial infarction. Stage I—histologic changes and processes directly induced by ischemia, stage II—beginning of inflammatory process, stage III and post-infarct left ventricular remodeling—irreversible histologic changes in the myocardium such as collagen secretion. ECM—extracellular matrix, HF—heart failure.

Post-infarction cardiac remodeling is a maladaptive, complex, and multifactorial process of regional and global structural and functional changes in the myocardium, and presents as a common complication of acute MI [16,131,132]. In most studies, the authors assessed changes which involved the left ventricle. Thus, in the sections below, we refer to remodeling of this chamber of the heart. This ultimately results in cardiac hypertrophy (CH) and heart failure (HF) [13,133]. Such modifications occur initially as a consequence of the loss of a viable myocardium and abrupt increase in loading conditions [134]. Then, the course of change depends on a plethora of determinants, including the size and location of the necrosis, the timing and efficacy of reperfusion, the exuberant inflammatory response, the increased wall stress in the border zone, and the remote myocardium, neurohormonal activation, and dysregulation of transcription [135,136,137,138,139,140]. All of these factors affect each other and create a vicious circle that leads to the gradual deterioration of heart function. There are numerous known causes that influence the course of this phenomenon after MI, and the most important clinically include arterial hypertension (HT), obesity, diabetes mellitus (DM), and ischemic heart disease, among others. The PI3K/Akt pathway plays a crucial role in post-infarction left ventricular remodeling (Figure 1c and Figure 2). Its activity provides cardioprotection and promotes the repair and healing of myocardial cells after MI [141]. Chen et al. showed that both PI3K and Akt activity were increased after MI in both human and mouse hearts. Moreover, the systemic as well as cardiac cell-specific inhibition resulted in greater cardiovascular risks and an increase in mortality of the mice. These results were associated with enhanced myocardial apoptosis and inflammation, reduced angiogenesis, and adaptive hypertrophy [142]. In the study undertaken by Feng et al., activation of the PI3K/Akt pathway by the PTEN inhibitor improved cardiac function 14 days after MI in mice. The reduced cardiomyocyte apoptosis promoted angiogenesis and activated the PI3K/Akt/vascular endothelial growth factor (VEGF) signaling pathway which resulted in significantly increased left ventricular fraction (LVEF), + dp/dtmax, and pressure–volume loops in the LV, as well as decreased left ventricular end-diastolic pressure (LVEDP). The contribution of the PI3K pathway to each of the stages of post-infarct remodeling and the impact of diabetes, is presented below.

### 4.1. Inflammation 

Injury of the cardiac cells and the extracellular matrix due to acute ischemia provides a strong systemic inflammatory response associated with increased production of pro-inflammatory cytokines [143]. These cytokines released during MI affect both the area of necrosis and the surrounding tissues and determine the course of post-MI left ventricular remodeling [144]. Both excessive and prolonged inflammatory reaction during MI are associated with an unfavorable prognosis for the patient. This process promotes myocardial damage, remodeling, and dysfunction of the left ventricle. As a large body of existing evidence from basic research and clinical trials indicates, this overactivity contributes to conditions such as chronic cardiac dilatation, left ventricular systolic dysfunction (LVSD), and HF [145,146,147,148]. There are multiple pathways involved in the course of post-MI inflammatory response, and PI3K/Akt signaling is one of the key ones [149]

C-reactive protein (CRP) is the most reactive serum protein of acute-phase inflammation and one of the most important prognostic biomarkers of atherosclerosis and cardiovascular disease (CVD) [150,151]. It affects the cell cycle and the inflammatory process of the cardiomyocytes [152,153]. CRP concentration increases significantly in the first hours of MI [131,154], and its level is a clinically important predictor of the course of left ventricular remodeling and patient prognosis [155,156]. Importantly, apart from inflammation, CRP is also a mediator of inflammation with prothrombotic and proapoptotic properties [157,158], which further worsens the prognosis of patients. Boras et al. noted that CRP in combination with a Notch-3 activator promotes angiogenesis in bovine aortic endothelial cells via the PI3K/Akt pathway [159]. Notably, pharmacological blockade of the PI3K/Akt survival pathway by LY294002 completely inhibited angiogenesis induced by CRP/Notch-3. Similarly, Chen et al. also noticed that CRP, through Akt activation, stimulates angiogenesis [160]. They revealed that the mechanism for this is the increase in the expression of vascular endothelial growth factor-A (VEGF-A) in activating hypoxia-induced factor-1α (HIF-1α) in adipose-derived stem cells (ADSCs). 

In contrast, Tanigaki et al. in their studies on mice (C57BL/6) showed that CRP attenuates insulin-induced Akt phosphorylation in endothelial cells [161]. Insulin promotes the protective cardiovascular endothelial functions by activating Akt, which then phosphorylates endothelial NO synthase (eNOS) in Ser1179, stimulating the production of cardioprotective NO [162]. CRP seems to impair this signaling and therefore increases the risk of CVD. Lee et al. noted that the reduction in the activity of the PI3K/Akt pathway mediated by CRP is related, inter alia, to the increase in the protein and mRNA levels of PTEN induced by CRP [163]. To sum up, the CRP protein and the PI3K/Akt pathway are undoubtedly strongly linked, and together play a significant role in regulating the inflammatory process. However, as the results of the direction of this interaction are inconclusive and there is insufficient research on these mechanisms during MI, there is a need for further research in this field.

In addition to CRP, the PI3K/Akt pathway also regulates the inflammation via other mechanisms. Parajuli et al. demonstrated that PTEN regulates the expression of important pro-inflammatory cytokines through the PI3K/Akt/IL-10/TNF-α signaling pathway and thus plays a significant role in post-MI remodeling of the myocardium in mice [164]. They indicated that inactivation of PTEN results in the induction of the inflammatory process after MI by reducing the expression of TNF-α and matrix metalloproteinase-2 (MMP-2), and by increasing the production of IL-10. In turn, the overexpression of PTEN can be observed during MI, which inhibits the PI3K/Akt pathway and thus causes the opposite effect on IL-10, TNF-α, and MMP-2 expression, and in addition increases leukocyte infiltration in the myocardium and increases cardiomyocyte mortality. Yuanji Ma et al. showed that the PI3K/Akt/Nrf2 pathway plays a role in attenuating inflammatory cells [165]. They showed that the stimulation of Akt phosphorylation with atorvastatin during angiotensin 2-induced oxidative stress activated Nrf2 in bone marrow-derived dendritic cells (BMDCs). As a result, it inhibited their maturation, which consequently promoted antioxidant and anti-inflammatory responses. Moreover, the use of the PI3K inhibitor LY2994002 abolished these positive effects [84,166,167]. 

It seems that the PI3K/Akt pathway may also play a role in other mechanisms of the inflammatory response. For example, the latest reports suggest that it variously mediates the signaling of interleukin-1 (IL-1), an important pro-inflammatory cytokine [168,169,170]. However, as these studies are sparse and have been performed on tissues other than the myocardium, it is not possible to draw direct conclusions. In recent years, clinical trials with IL-1 inhibitors (e.g., with canakinumab and anakinra) have been promising, and suggest a reduction in the incidence of cardiovascular complications among post-MI patients [171,172,173]. Therefore, more research is needed to better understand the mechanisms of these interactions in the context of PI3K/Akt.

The drugs used after MI onset can modulate the inflammation process. Among numerous positive effects of statin therapy, they can inhibit the expression of pro-inflammatory cytokines, such as TNF-α, by direct activation of nuclear factor (NF)-κBα [174]. Moreover, the downexpression of the PI3K/Akt pathway can enhance the immune system activation [174,175]. Thus, regulation of the PI3K/Akt pathway can be used to reduce the inflammation and infiltration of the myocardium by immune cells, consequently improving post-MI left ventricular remodeling [176].

Taken together, the results of experimental and clinical trials on anti-inflammatory strategies targeting the PI3K/Akt pathway in MI patients are promising regarding mitigating the effects of infarction. For this reason, there is undoubtedly a need for further research, both on existing and potentially new therapies.

### 4.2. Autophagy

Autophagy is an evolutionarily conserved cellular process of degradation of both unnecessary and dysfunctional components of the cell, including mitochondria and long-lived macromolecules. Moreover, it ensures the availability of energy substrates and reduces oxidative stress that would otherwise promote cell death [177,178]. Therefore, it is an important regulator of cardiac homeostasis and function. Autophagy is a lysosome-dependent process that utilizes double-membrane structures (autophagosomes) as a form of intracellular transport, and enables the cell to degrade and recycle its components [179,180]. Autophagy preserves the structure and function of the cardiomyocytes under baseline conditions, and during infarction and may be induced by various factors associated with MI, such as nutrient deprivation, hypoxia, ROS, damaged organelles, and protein aggregates [181,182,183]. It could be of high importance in the peri-infarct zone. There are multiple significant mechanisms of autophagy regulation in the cardiomyocytes, the disruption of which impairs autophagy activation and exacerbates myocardial injury [183,184,185]. The PI3K/Akt pathway plays a significant role in some of them, as we describe in this section.

One of the key pathways is the PI3K/Akt/mTOR signaling pathway. mTORC1 is an important serine/threonine kinase and is an upstream repressor of autophagy. Kim et al. observed that mTORC1 in vitro inhibits autophagosome formation by phosphorylation of unc-51-like autophagy activating kinase 1 (Ulk1) at Ser757 [186]. In addition, mTORC1 also acts at the gene level. As Martina et al. noticed, it has been observed to inhibit the transport of nuclear transcription factor EB (TFEB), a regulator of autophagy and lysosomal biogenesis, and thus downregulates the transcription of specific autophagy-related (Atg) genes [187]. Under physiological conditions, autophagy is activated in cells under stress. In contrast, Sciarretta et al. revealed that Akt phosphorylates diverse substrates during MI, indirectly causing forced activation of mTORC1 in the myocardium [184,188]. This sequence of events leads to the inhibition of autophagy and fiercely intensifies ischemic injury [184]. Interestingly, besides Akt, mTORC1 can also be regulated via other cellular signaling pathways such as MAPK/ERK, JNK, and Wnt signaling [189,190,191].

Moreover, PI3K can also inhibit autophagy directly by phosphorylating Beclin-1, a core component of Beclin 1-PI3KC3 complex, a lipid–kinase complex playing a crucial role in autophagosome nucleation [192]. Phosphorylation of Beclin-1 enhances its interaction with BCL-2, and this Beclin 1–BCL-2 interaction not only inhibits autophagy but also dissociates BCL-2 from Bax, thereby activating Bax and stimulating apoptosis. Matsui et al. revealed that autophagy via Beclin-1 is particularly activated during reperfusion by the mass-produced ROS during that period [185]. In addition, in mice (C57BL/6J) studies, they showed that mice with systemic heterozygous Beclin 1 deletion display significantly reduced autophagy and ischemic injury. Interestingly, Beclin 1 can both positively and negatively regulate autophagy, hence the optimum concentration range of this molecule is needed for autophagy to function efficiently [193]. 

Via both the abovementioned pathways, PI3K/Akt signaling negatively modulates autophagy and therefore has a protective effect on the heart. Yan et al. also observed that in the pig heart, MI-induced autophagy could be a homeostatic mechanism which inhibits apoptosis and hence limits its destructive effects [182]. Taken together, stimulation of autophagy may have a protective effect on the heart [194,195], which may be another potential target of the therapy.

In addition, by regulation of autophagy and apoptosis, the activity of the PI3K/Akt/mTOR pathway contributes to myocardial remodeling following MI [196]. It may suppress the progression of HF and cardiac hypertrophy (CH). Pharmacological inhibition of autophagy (e.g., with rapamycin) exacerbates cardiac dysfunction and dilation in the chronic phase of MI [197,198]. Nevertheless, the activity of autophagy seems not to be sufficient to control the quality of proteins and organelles during MI. Consequently, misfolded proteins accumulate, and mitochondrial dysfunction develops in post-MI hearts. Regulation of key proteins in this pathway has been shown to reduce infarct size [199]. These observations suggest that autophagy may potentially contribute to the heart’s healing process by mechanisms such as activation of the repairing process, angiogenesis, or promotion of cardiac regeneration. Thus, modulation of autophagy could be used to alleviate HF and CH after MI.

### 4.3. Fibrosis

Myocardial fibrosis is a repair process which involves multiple modifications in the interstitial myocardial collagen network, which results in the impairment of the cardiac structure and function. The replacement of the necrotic myocardium by connective tissue is the main mechanism of fibrosis during post-infarction left ventricular remodeling [200]. As cardiomyocytes are displaced with fibrous tissue, many detrimental effects occur. These include excitation–contraction coupling and the systolic and diastolic function of the heart, which can lead to HF [201]. The PI3K/Akt pathway can regulate fibrosis in several ways. Cardiomyocyte apoptosis initiates the fibrotic response, and this process may involve both immune modulation and paracrine signaling [202]. Therefore, the PI3K/Akt pathway performs a protective effect in post-infarction fibrosis by inhibiting the death of cardiomyocytes [203]. 

Activation of the PI3K/Akt pathway may significantly increase cardiac dysfunction when its inhibition has the reverse effect, and increases fibrosis in the infarcted area [66]. Indeed, it has been proved that the administration to animals of one of the PI3K/Akt inhibitors such as wortmannin or LY294002 reduces fibrosis and post-infarct remodeling [121]. It seems that Akt agonists or PTEN inhibitors may show a positive effect on post-infarct remodeling and improve a patient’s outcome, but all the conducted studies have focused on the oncologic utility of this group of substances [65,204]. It seems that they may be used as a part of antifibrotic therapy after MI, but the assessment of their safety and tolerance is needed [204].

In contrast, Zhao et al. in their research noted that the active PI3K/Akt pathway may promote myocardial fibrosis [205]. The authors showed that the activation of this pathway by long noncoding RNA (LncRNA) myocardial infarction-associated transcript (MIAT) promotes the expression of inflammatory factors in the myocardium. Conversely, when LncRNA MIAT was silenced, the levels of vascular endothelial growth factor (VEGF), Akt, and PI3K levels were significantly downregulated. In addition, as this silencing resulted in a reduction in collagen expression, regulation of these interactions could potentially improve cardiac repair and consequently ameliorate HF.

One of the initial processes involved in myocardial remodeling is the activation of fibroblasts. It was shown that the response of cardiomyocytes to fibroblast growth factor (FGF) can be impaired after MI. The expression of dysregulated proteins can promote activation of fibroblasts and cause the accumulation of collagen types I and III, which are the major fibrins of the myocardial collagen matrix. This post-infarct remodeling of the interstitium, mainly of the left ventricle, is a major cause of cardiac hypertrophy and can be responsible for the development of HF after MI. It seems that PI3K/Akt activity may be involved in these processes [109]. Some drugs which are prescribed for patients after MI have an antifibrotic effect. ACEIs and mineralocorticoid receptor antagonists (MRAs) are well-known drugs that present this effect. However, there are no data showing that the PI3K/Akt pathway is directly involved in this process. However, it has been shown that the stimulation of β-receptors can stimulate neonatal rat cardiac fibroblasts to engage in protein synthesis with a simultaneous increase in PI3K activity. This process may be involved in collagen production after MI, so it seems that the administration of β-adrenolytics can reduce the activity of fibroblasts and improve cardiac function among patients not only due to the positive-inotropic and anti-arrhythmic effect of these group of drugs [206]. 

### 4.4. Cardiac Hypertrophy 

Hypertrophy of the myocardium occurs as a response to stress stimuli such as infarction and is an important component of left ventricular remodeling after MI. Such structural changes are associated with increased cardiomyocyte apoptosis and fibrosis and result in contractile dysfunction, dilatation, and consequently, the development of HF [207,208]. There are numerous triggers of cardiac hypertrophy, such as mechanical stress and humoral stimulation, which can lead to multiple metabolic responses [209]. The PI3K/Akt signaling pathway and its interaction with its downstream effectors are altered and play a key role in regulating this process [210].

Short-term activation of Akt promotes physiological hypertrophy, and despite leading to mild myocardial enlargement, it has a cardioprotective effect [211] by attenuating damage to ischemia in endothelial cells [212]. Nevertheless, long-term Akt activation induces pathological hypertrophy and HF [213]. In this case, PI3K/Akt increases angiogenesis, which was mentioned in the previous section, but these blood vessels are, however, unorganized and reminiscent of tumor vasculature [212]. In addition, Zhao et al. [205] in their research on human cardiac fibroblasts (HCF) and rats (C57BL/6) have noticed that the activation of the PI3K/Akt pathway by myocardial infarction-associated transcript (MIAT) promotes myocardial fibrosis and the expression of various inflammatory factors such as IL-1β, IL-6, and TNF-α mRNA, and numerous proteins that collectively contribute to the occurrence of HF. Muting their MIAT reduced the incidence of HF. Meng et al. [214], on the other hand, found that the PI3K/Akt pathway in mice (C57BL/6J) is involved in the development of hypertrophy by means of yet another molecule, the aforementioned BCL-2, as it maintains cardiomyocyte survival.

All the mentioned reports suggest that PI3K/Akt signaling plays a significant role in post-infarction myocardial hypertrophy, and that adequate regulation of this pathway could potentially be an important therapeutic target to reduce the incidence of HF. However, as these studies were performed only on an animal model, the effectiveness of this regulation needs to be evaluated in clinical trials.

### 4.5. Angiogenesis

As a constant supply of blood to cardiomyocytes is of fundamental importance for their proper functioning, an adequate vascularization system in the myocardium is essential. Angiogenesis is a complex process consisting of endothelial proliferation, cell migration, and eventually the formation of blood vessels. It has been noticed that PI3K/Akt plays a pivotal role in activating angiogenesis and in forming collateral circulation via, among others, regulating the transcription and expression of proangiogenic cytokines such as vascular VEGF, angiopoietin-1 (Ang-1), and bFGF (which in addition reverse-stimulates Akt phosphorylation) [215,216]. As these growth factors stimulate the formation of blood vessels, they improve the supply of oxygen and nutrients to the heart. Consequently, this activation has the potential to both rescue the myocardium at early stages after AMI and prevent subsequent ischemic-related heart failure [217].

Moreover, overexpression of PTEN (PI3K/Akt inhibitor) attenuates these positive effects and, for example, inhibits angiogenesis and contributes to thrombosis by inducing endothelial dysfunction [218,219]. 

Research shows that the downregulation of PTEN could promote angiogenesis through increasing the expression of VEGF as well as reinforcing the signal transduction of the VEGF-binding cell [220,221]. In addition, such PTEN inhibition results in an increase in the expression of the cluster of differentiation 31 (CD31), also known as platelet endothelial cell adhesion molecule (PECAM-1). This molecule participates in the creation of adhesive interactions between endothelial cells and adhesion receptors. This makes it possible to mitigate the impairing effect of MI on capillary density [65].

As angiogenesis has such a cardioprotective potential in the early stages after MI, therapies focused on the PI3K/Akt pathway have become a novel treatment strategy for patients after infarction [217,222]. 

### 4.6. Conduction Disturbances

Post-infarction severe arrhythmias, especially ventricular arrhythmias (VA), are the leading cause of death in MI patients. The most common arrythmias observed in patients with MI are ventricular tachycardia (VT, 17–21%), advanced and complete heart block (23–35%), ventricular fibrillation (VF, 24–29%), and atrial fibrillation (AF, 11–20%) [223]. Such arrhythmias are the result of sympathetic remodeling, a phenomenon of disturbance of the spatial distribution and density of the myocardial sympathetic innervation caused by denervation in the infarcted zone and hyperinnervation in the infarcted border zone [224,225]. Such a sympathetic remodeling may cause electrophysiological disturbances and increased heterogeneity of noradrenergic transmission, and consequently increase the risk of dangerous arrhythmias [226]. The key cytokine involved in sympathetic remodeling following MI is the nerve growth factor (NGF), part of the NGF/TrKA/PI3K/Akt pathway that regulates neuronal plasticity and survival [227,228]. PI3K/Akt signaling is essential for NGF and other neurotrophic activities [26]. Li et al. showed that the appropriate regulation of the PI3K/Akt pathway by inhibiting its excessive activity reduces the abnormal excitability, automatism, and conductivity of residual myocytes, which result in the initiation of arrhythmia after myocardial infarction in rats [229,230]. Taken together, targeting the NGF/TrKA/PI3K/Akt pathway has the potential to regulate factors associated with sympathetic remodeling and thus potentially reduce the incidence of post-MI arrhythmias. [230]. However, these positive effects were only noticed in preclinical trials, and further investigation is required in this field. 

## 5. Diabetes

Experimental studies on both in vitro and animal models of DM indicates the higher susceptibility of cardiomyocytes to IRI. Infarct size in animal models seems to be enlarged, however some results are contradictory [231]. Moreover, DM is associated with significantly higher mortality one year after ACS compared with non-DM patients, and this partially results from more severe damage of the myocardium [232]. Clinical studies show that the median infarct size is larger in diabetic patients and reperfusion is impaired [233,234]. Moreover, we observe the altered expression of the PI3K/Akt pathway in the diabetic heart. Preclinical studies show that this may play a crucial role in cardiac susceptibility to IRI and impaired cardioprotection in diabetic patients [235].

The cardiomyocytes of the adult rat are exposed to increased intracellular oxidative stress and apoptosis in hyperglycemic and free fatty acid conditions. Treatment with ghrelin decreases the apoptosis rate by 25–44% and is associated with increased activation of the PI3K/Akt pathway. This promotes phosphorylation of both Akt and ERK1/2 and the nuclear translocation of NFκB, which promotes anti-apoptotic BCL-2 and Bcl-xL expression [236]. The antiapoptotic effect of ghrelin is reversed by treatment with wortmannin (a PI3K inhibitor). Therefore, hyperglycemia impairs the antiapoptotic effect of PI3K/Akt in cardiomyocytes and its promotion can protect cells. Activation of PI3K/Akt is also diminished in the hearts of Sprague Dawley rats with streptozotocin (STZ)-induced diabetes. It decreases the expression of p308-Akt, p473-Akt, p136-BAD, Bcl-2, and the Bax/Bcl-2 ratio when compared with the non-diabetic group. Treatment with luteolin increases the expression of fibroblast growth factor receptor 2 (FGFR2) and leukemia inhibitory factor (LIF), which results in the activation of the PI3K/Akt pathway. It is associated with reduced cardiomyocyte apoptosis, diminished infarct size, and preserved cardiac function after IRI [237]. Chen et al. showed that Sprague Dawley rats with DM induced by a high-fat diet combined with low-dose STZ have a decreased level of Akt phosphorylation, and it is not enhanced by IRI. In opposition, activity of the PI3K/Akt pathway significantly increases in non-DM rats, which is connected with smaller infarct size and a lower apoptosis rate when compared with the DM group after IRI. Inhibition of JNK activity can partially restore the function of the PI3K/Akt pathway, which results in decreased apoptosis and improved cardiac function in DM rats [238]. Using a similar model, An et al. showed that apelin treatment can increase PI3K activity in DM rats and this leads to increased expression of its downstream effector, impairing the Notch1/Hes1 pathway. This results in decreased expression of iNOS and an enhanced eNOS level, alleviated cardiomyocyte apoptosis and lower infarct size after IRI, and improved cardiac function 6 weeks after IRI [239]. The impact of the PI3K/Akt signal on the Notch1/Hes1 pathway and its beneficial effect on the diabetic myocardium after IRI was also presented by other researchers [240].

The impaired cardioprotective effect of the PI3K/Akt pathway in the diabetic heart also results in non-efficient ischemic conditioning. Tsnag et al. showed that hearts of diabetic Goto–Kakizaki (GK) rats require more cycles of preconditioning in order to experience a significant reduction in the infarct size than non-diabetic Wistar rats [241]. Even though one cycle of IC induces phosphorylation of Akt (Ser 473) compared with the control GK hearts (24.7 ± 5.0 arbitrary units (AU) vs. 6.2 ± 0.9 AU, respectively; *p* < 0.05) it is not related with a reduction in the infarct size. However, three cycles of IC induce higher levels of p-Akt (42.4 ± 3.2 AU; *p* < 0.01) and results in decreased infarct size compared to the control hearts (20.8 ± 2.6 vs. 46.6 ± 5.2%; *p* < 0.01). Inhibition of PI3K signaling with LY294002 reduced the p-Akt level (20.8 ± 2.1 AU; *p* < 0.01) and abrogated the infarct size (44.9 ± 6.4%; *p* < 0.01). In non-diabetic Wistar rats, one cycle was sufficient to reduce the infarct size and significantly increase Akt phosphorylation (72.4 ± 7.5 and 74.2 ± 9.1 vs. 37.1 ± 5.7 AU, respectively; *p* < 0.01). Therefore, the threshold for cardioprotection provided by activity of the PI3K/Akt pathway is elevated in diabetic myocardium. DM inhibits preconditioning and postconditioning, affecting PI3K/Akt on many levels [122,242]. For example, activation of STAT3 is strongly reduced in STZ-induced diabetic rats, which results in the inhibition of PI3K/Akt activity and decreased cardioprotection [243]. This effect may be caused by high glucose-dependent oxidative stress, which abrogates remifentanil preconditioning in STZ-induced diabetic rats [244]. DM decreases phosphorylation of Akt on ser473 and STAT3 on Tyr705 and ser727, without influencing total Akt and total STAT3 expression at baseline. Moreover, it can be restored by antioxidant treatment with N-acetyl cysteine (NAC), by improving Akt and STAT3 activation, and by facilitating the cross talk between the PI3K/Akt and JAK2/STAT3 signaling pathways, resulting in attenuated myocardial IRI and post-MI LV dysfunction. Furthermore, sevoflurane postconditioning can be diminished by the inactivation of T-LAK cell-originated protein kinase (TOPK). This protein kinase can inactivate PTEN, thus inducing PI3K/Akt activity. Alteration of this pathway was observed in STZ-induced diabetic mice and cardiac cells exposed to high glucose in vitro [245]. Moreover, selective inhibition of PTEN may preserve the cardioprotective effect of ischemic postconditioning in STZ-induced diabetic rats [246]. Other studies indicate that impairment of PI3K/Akt signaling results in decreased activity of its downstream effector, GSK-3β, leading to ineffective cardioprotection [247,248]. 

Diabetes mellitus is also a strong predictor of post-MI HF development. Among patients with first anterior MI, DM was associated with a greater risk of cardiovascular death or rehospitalization for HF [249]. Other authors have also observed an increase in the left atrial volume index (LAVi) as well as left atrial enlargement at 20-month follow-up [250]. These studies indicate the long-term elevation in LV diastolic pressure post-MI among diabetic patients, which may be caused by adverse remodeling and might mediate the risk. DM is also an independent predictor of the higher risk of rehospitalization of HF among patients who underwent PCI ((HR) 1.576, *p* = 0.010) [251]. In a study by Akashi et al., DM was associated with a higher risk of major adverse cardiovascular events (MACEs) ((HR) 2.79, *p* = 0.017) and HF hospitalization ((HR) 3.62, *p* = 0.023) after STEMI, and left ventricular remodeling at the baseline was an independent risk factor [252]. Moreover, Yang et al. showed that insulin resistance is also a strong predictor of post-MI HF. They observed greater LV dilation after STEMI among patients with impaired fasting glucose (IFG), impaired glucose tolerance (IGT), and high HOMA-IR levels, who underwent primary percutaneous coronary intervention and were followed up for 12 months [253]. The mechanisms underlying the higher risk of HF after MI in diabetic patients remain unknown. However, we can observe similar changes in animal studies, and the PI3K/Akt pathway may be an important mediator.

Multiple studies show aggravated left ventricular remodeling and HF after MI in experimental diabetes. Hyperglycemia increases the apoptosis rate of cardiomyocytes and induces cardiac fibrosis, leading to left ventricular enlargement and dysfunction [254,255]. Moreover, Backlund et al. showed that the number of apoptotic cells in the border zone of infarction as well as in the non-infarction site is significantly higher among diabetic rats at 12 weeks after MI. Moreover, Thakker et al. observed an altered inflammatory response and healing process leading to adverse cardiac remodeling in diet-induced obesity and insulin resistance after myocardial IRI [256].

Modification of the PI3K/Akt expression may play an important role in lower survival rate, worse LV function, and aggravated myocardial fibrosis. Vahtola et al. showed that pronounced cardiomyocyte hypertrophy, increased fibrosis, and cardiomyocyte apoptosis in diabetic rats 12 weeks after MI were associated with decreased Akt activation and increased nuclear localization of FOXO3a (forkhead box O3) [257]. These changes resulted in the increased expression of PTEN and the inhibition of the cardioprotective properties of the PI3K/Akt pathway. Moreover, Akt played a key role in mediating the cardiac remodeling induced by EGFR stimulation in diabetic mice. This results in ROS generation and cardiac damage which can be decreased by EGFR inhibitors [258]. 

It is not only the diabetic state and hyperglycemia that can impact the activity of the PI3K/Akt pathway and influence the infarction size and post-infarction left ventricular remodeling, but also common antidiabetic drugs can do so.

Metformin has cardioprotective effects which are mediated by many pathways including PI3K/Akt. Bhamra et al. showed that rats receiving metformin had reduced infarct size in comparison with the placebo group (35 +/– 2.7% vs. 62 +/– 3.0%, *p* < 0.05). This protective effect was also observed in the animal model of DM (43 +/– 4.7% metformin vs. 60 +/– 3.8% control, *p* < 0.05) [259]. The authors observed that it was accompanied by a significant increase in Akt phosphorylation. Furthermore, the addition of the PI3K inhibitor abolished the metformin-induced Akt phosphorylation and the protective effect [259]. Moreover, metformin can be effective in the treatment of diabetic cardiomyopathy. In an animal study, administration of metformin significantly reduced the expression of caspase-1 and nucleotide-binding domain, leucine-rich-containing family and pyrin domain-containing-3 (NLRP3) inflammasome, which resulted in the inhibition of pyroptosis as a consequence. Moreover, this antidiabetic drug can inhibit the expression of mTOR and reduce the activity of autophagy [260]. All this evidence shows that metformin has a cardioprotective effect.

Insulin activates PI3K and Akt which provide cardioprotection via the subsequent activation of many eNOS, mTOR, and reduction in ROS production. Administration of insulin stimulates eNOS to produce NO, one of the most important vasoprotective substances. In addition, it was observed that wortmannin (a PI3K inhibitor) can abolish this positive effect [261]. Another basic study showed that insulin can reduce apoptosis and necrosis by suppressing ROS production, which was also mediated by the PI3K/Akt pathway [262]. Moreover, insulin can activate mTOR kinase, which may inhibit inflammation and fibrosis, responsible for adverse left ventricular remodeling and hemodynamic dysfunction [263]. It seems that activation of the PI3K/Akt pathway is one of the main targets of insulin and the mechanism of the cardioprotective effect of this antidiabetic drug. 

SGLT2 inhibitors are primary antidiabetic drugs which caused a revolution in the treatment of HF. The administration of these drugs significantly reduces mortality among patients with HF, even without diabetes [264]. The mechanism of action is still not fully understood. It seems that some of the positive effects can be mediated by the PI3K/Akt pathway. Hasan et al. showed that SGLT2 inhibitors can attenuate oxidative stress induced by isoprenaline by reducing inflammation [265]. They revealed that administration of canagliflozin can reduce fibrosis of the Long–Evans rat heart. It was associated with diminished reduction in Akt phosphorylation by the SGLT2 inhibitor. The authors simultaneously noted numerous changes in the expression of other proteins such as AMPK and eNOS. The significance of the PI3K/Akt pathway in cardioprotection should be confirmed. 

Additionally, the GLP-1 mimetics can attenuate adverse left ventricular remodeling. Robinson et al. showed that the infusion of exendin-4 for 4 weeks after MI can improve survival and protect against cardiac dysfunction [266]. A broad spectrum of its activities decreases cardiomyocyte apoptosis, attenuates fibrosis, and inhibits myocardial inflammation by the modulation of the Akt/GSK3β pathway, among others. 

To summarize, restoring the correct function of the PI3K/Akt pathway in the diabetic heart is a potential target for diminishing infarct size and improving cardiac function after IRI. The PI3K/Akt pathway may also play a significant role, and its dysregulation results in adverse remodeling, leading to cardiac dysfunction. Pharmacological treatment may improve the outcome for patients after MI, affecting the cardioprotection mediated by this pathway. This could improve the outcome and reduce post-MI mortality in diabetic patients.

## 6. MicroRNA

MicroRNAs are a short non-coding RNAs of about 22 nucleotides that can mediate gene silencing by guiding Argonaute (AGO) protein to target sites in the 3′ untranslated region of the mRNAs. MiRNAs can be secreted out of a cell and mediate cell communication in a paracrine and endocrine manner. They can be transported to target cells by binding to proteins, including AGO, or within extracellular vesicles t(EVs) [267]. Moreover, miRNA levels in circulation differ in various pathological states, including myocardial infarction and diabetes. This can be associated with the increased vulnerability of the heart to ischemic injury or the adverse post-MI remodeling of the left ventricle [268]. Below, we present miRNAs that can regulate cardiac cells’ metabolism and target the PI3K/Akt pathway activity (Table 1), and describe the most interesting examples.

Expression of miR-21 is significantly increased in hearts subjected to IRI after 2 and 7 days of reperfusion compered to sham-treated hearts [269]. MiR-21 regulates activity of the PI3K/Akt pathway by targeting the 3′-UTR of a PTEN mRNA. Its inhibition results in elevated phosphorylation of Akt, which causes induction of matrix metalloprotease-2 (MMP-2) in cardiac fibroblasts in vitro [269]. MMP-2 is involved in the remodeling of the extracellular matrix and may lead to the impaired ventricular contraction [270]. Moreover, mir-21-induced activity of Akt signaling decreases the Bax/Bcl-2 ratio and expression of caspase-3, which can inhibit apoptosis of human H9C2 cardiomyocytes and lead to a reduction in the infarct size in the SD rats IRI model [271,272]. Furthermore, miR-21 induces the Akt/mTOR signaling pathway, which inhibits autophagic activity and alleviates apoptosis of H9C2 cells [273].

Interestingly, inhibition of PTEN by miR-21 mediates the cardioprotective effect of ischemic postconditioning and results in alleviated myocardial apoptosis, decreased infarct size and improved left ventricle function in mice [274]. Loss of miR-21 reverses all these beneficial effects and can also reverse the cardioprotective effect of isoflurane preconditioning, which is associated with increased phosphorylation of Akt and eNOS [275]. Moreover, mir-21 can be also transported inside EVs and therefore be a potential MI regenerative therapy target [276]. Interestingly, dysregulation of miR-21-5p within EVs from heart failure patients causes loss of their beneficial effects and impairs their ability to promote both angiogenesis and cardiomyocyte proliferation. In contrast, they exacerbate left ventricle remodeling and decrease LVEF in mice 3 weeks after intramyocardial injection [277]. Restoration of miR-21 expression in the EVs of HF patients rescues their cardioprotective effect. Therefore, miR-21 plays an important role in regulation of the PI3K/Akt signal in cardiomyocytes and may impact the heart’s ischemic injury and remodeling. 

MiR-126 plays an important role in angiogenesis and vascular homeostasis. It targets the 3′-UTR of Phosphoinositide-3-Kinase Regulatory Subunit 2 (PIK3R2), which is a gene encoding regulatory subunit p85β. Its inhibition results in up-regulated activity of PI3K and Akt signals. Song et al. reported that expression of miR-126 may be triggered by HIF-1α in endothelial cells of post-MI hearts, which can affect hypoxia-induced tube formation and angiogenic signaling. Adaptive exercise training seven days after MI induction can improve myocardial angiogenesis and cardiac function, increasing LVSP, +dp/dt max, and decreasing LVEDP compared with the MI SD rat group. This effect is associated with increased expression of HIF-1α and miR-126, and reduced expression of PIK3R2 and Sprouty-related protein 1 (SPRED1). Moreover, all these results can be attenuated by HIF-1a inhibitor. Further study on human umbilical vein endothelial cells (HUVECs) suggests that miR-126 may be involved in tube formation under hypoxia through the PI3K/AKT/eNOS pathway [278]. PIK3R2 is also a negative regulator of the VEGF pathway, and its inhibition by miR-126 promotes the function of endothelial progenitor cells (EPC) under hypoxic conditions. Overexpression of miR-126 enhances viability, migration and tube-forming ability of EPCs, which can play important role in vascular integrity and repair after MI [279].

Both obesity and diabetes downregulate miR-126 expression level (Table 1). Gomes et al. showed that this is associated with decreased capillary density in skeletal muscles of obese Zucker rats. Interestingly, exercise training restores miR-126 expression, and promotes angiogenesis by decreasing PI3KR2 activity and inducing VEGF and eNOS. It results in enhanced skeletal muscle capillary rarefaction [280]. Furthermore, miR-126 is downregulated in the EPCs from diabetic patients and impair their function by targeting SPRED1 and impairing Ras/ERK/VEGF and PI3K/Akt/eNOS signal pathways [281]. Moreover, downregulation of miR-126 may result in increased apoptosis of vascular endothelial cells and elevated myocardial susceptibility to IRI [282,283]. Therefore, miR-126 can play an important role in regulation of PI3K/Akt signal transduction during myocardial infarction and remodeling. It is a potential post-MI prognostic factor and target for miRNA-based therapeutic interventions for DM complications. Interestingly, the circulating level of miR-126 is downregulated in diabetic patients, what can be associated with the impaired cardioprotective function of the PI3K/Akt pathway. Furthermore, the above effects on angiogenesis may be also mediated by EVs-derived miR-126, and exosomes from miR-126-overexpressing mesenchymal stem cells (MSCs) may be a promising therapeutic strategy to promote angiogenesis and wound repair [284]. 

MiR-145 can also play a cardioprotective role, attenuating both myocardial ischemia–reperfusion injury and post-infarct remodeling via targeting the PI3K/Akt pathway. It inhibits cardiac cells’ apoptosis, ROS activity and increases cells’ viability under hypoxic conditions in vitro. These effects are positively related to the activation of PI3K/Akt and expression of SGK1 (serum- and glucocorticoid-regulated kinase (1)—a downstream effector of PI3K associated with cell survival [285]. Moreover, miR-145 promotes autophagy and reduces the infarct size in AMI models in vivo via targeting FRS2 (fibroblast growth factor receptor substrate (2) and activating the PI3K/Akt/mTOR pathway [286,287]. Additionally, miR-145 can inhibit post-infarct remodeling. It reduces both LV systolic and diastolic dimensions and improves ejection fraction and +dP/dt in rabbit MI model [286]. Moreover, it attenuates post-infarct fibrosis and prolonged action potential duration via activation of Akt/CREB cascades, which leads to reduced response to β-adrenergic stimuli [288]. On the other hand, miR-145 has been shown to attenuate fibrosis and improve cardiac function via suppressing the AKT/GSK-3β/β-catenin signaling pathway in fibroblasts [289]. Nevertheless, miR-145 seems to have beneficial effect on cardiac repair, and its augmentation can be an attractive target for preventing both cardiac IRI and remodeling after MI. A study on 246 patients with first STEMI who underwent successful PCI showed that circulating miR-145 on day 5 post-MI is independently associated with MACE (HR 7.174, 95% CI 4.208–12.229 [*p* < 0.0001] and cardiac death (HR 5.628, 95% CI 1.990–15.911 [*p* = 0.0012]) during the one-year follow-up period [290]. Moreover, miR-145 levels peak at day 1 post-AMI, which negatively correlates with the EF (Spearman ρ = 0.65, *p* < 0.0001) [291]. 

**Table 1:** A list of microRNAs which can impair PI3K/Akt signal activity and potential mechanisms of their impact on ischemia–reperfusion injury and post-infarction remodeling.

miRNA	DM2	Ref.	The Potential Regulatory Mechanism
miR-19	-	-	MiR-19a protects H9C2 cardiomyocytes against H/R-induced apoptosis by inhibiting PTEN [292]; MiR-19b promotes NRCFs proliferation and migration by targeting PTEN [293].
miR-21	up *	[294,295,296,297]	Targets PTEN expression and promotes adverse ventricular remodeling by induction of MMP-2 in cardiac fibroblasts [269], alleviates cardiomyocytes apoptosis and reduces infarct size through decreasing Bax/Bcl-2 ratio and caspase-3 expression [271], decreases cardiomyocytes autophagy [273].
miR-34	up	[294]	Inhibition of miR-34a attenuates MI-induced LV remodeling in mice and induces Akt phosphorylation [298]; activates PI3K/AKT pathways via up-regulating ZEB1 in cardiomyocytes and attenuates hypoxia-induced injury [299]; protects H9C2 cardiomyocytes from high-glucose-induced injury [300].
miR-122	up	[295,301]	Aggravates oxygen–glucose deprivation and reperfusion apoptosis of H9C2 cardiomyocytes inhibiting AKT/GSK-3β/β-catenin and AKT/mTOR pathway signaling [302,303];
miR-126	down	[295,304,305,306,307,308]	Targets PIK3R2 and SPRED1 expression, resulting in elevated activity of the PI3K/Akt signal and improved angiogenesis, left ventricle function after MI, and alleviated apoptosis of both endothelial cells and cardiomyocytes [278,279,280,281,282,283,284]
miR-130	up	[295,309,310]	Attenuates LV dysfunction and remodeling after MI targeting PTEN and increasing activity of Akt [311]
miR-145	down	[312]	Inhibits cardiac cells apoptosis and ROC activity by enhancing the PI3K/Akt and SGK1 activity [285], promotes autophagy and reduces myocardial infarct size targeting FRS2 and inducing PI3K/Akt/mTOR activity [286,287], attenuates fibrosis via activation of the Akt/CREB and suppression of the AKT/GSK-3β/β-catenin pathways [288,289].
miR-155	up	[313,314]	Targets IKKi expression decreasing its cardioprotective role in activating Akt and NF-κB, independent of the PI3K, and enhances cardiac hypertrophy [315], inhibits the AKT/CREB pathway signal and impairs the left ventricle function [316].
miR-223	up	[310,317,318]	Inhibits angiogenic function of CMECs by decreasing the PI3K/Akt signal activity [319], regulates cardiac hypertrophy by modulating the p-Akt activation [320], and mediates cardiac fibrosis after MI targeting RASA1 expression, which promotes MEK1/2, ERK1/2 and AKT phosphorylation [321].
miR-320	up	[295,306]	Increases vulnerability of cardiomyocytes to hypoxia/reoxygenation injury targeting expression of Akt3 [322].
miR-375	up	[323,324]	Exacerbates inflammation and cardiomyocyte apoptosis, decreases angiogenesis and impairs the LV function after MI by a reduction in PDK-1 expression, which results in decreased Akt Thr-308 phosphorylation [325,326].

(DM—diabetes mellitus, *—indicates a contradictory finding where the miRNA was found to be down-regulated in at least one study). Akt—protein kinase B, Bax—Bcl-2-associated X protein, Bcl-2—B-cell lymphoma 2, CMECs—cardiac microvascular endothelial cells, CREB—cAMP response element-binding protein, ERK1/2—extracellular signal-regulated kinase1/2, FRS2—fibroblast growth factor receptor substrate 2, GSK-3β—glycogen synthase kinase 3 β, LV—left ventricle, MEK1/2—mitogen-activated protein kinase kinase1/2, MI—myocardial infarction, MMP-2—metalloproteinase 2, mTOR—mammalian target of rapamycin, NF-κB—nuclear factor κ-light-chain-enhancer of activated B cells, NRCFs—neonatal rat cardiac fibroblasts, IKKi—inducible IkB kinase, H/R—hypoxia/reoxigenation, p-Akt—phosphorylated Akt, PDK-1—phosphoinositide-dependent kinase-1, PI3K—phosphoinositide 3-kinase, PIK3R2—phosphoinositide 3-kinase regulatory subunit 2, PTEN—phosphatase and tensin homolog, RASA1—Ras p21 protein activator 1, ROC—Ras of complex proteins, SGK1—serum and glucocorticoid-regulated kinase 1, SPRED1—Sprouty-related EVH1 domain containing 1, Thr-308—threonine 308, ZEB1—zinc finger E-box binding homeobox 1.

MiRNAs may play and important role in the modulation of PI3K/Akt activity in the heart. They may serve as potential biomarkers and prognostic factors of the infarct size and adverse post-MI remodeling. Moreover, miRNAs can mediate the effect of DM on the impaired PI3K/Akt signaling in the heart.

## 7. Further Perspectives

The PI3K/Akt pathway plays an important role in the survival and function of cardiomyocytes. It seems that this pathway may be an ideal target to protect the myocardium against most of the post-infarct changes described above. In addition, PI3K and other components of the described pathway are among the essential links in the inflammatory response after ischemia. Substances activating this pathway (e.g., SC79) are at an advanced stage of development in basic research, but still the results are not conclusive [327,328,329]. It seems that these drugs can be useful in patients after MI and may reduce the risk of post-infarct HF. However, there are no sufficient in vivo analyses or clinical trials to test the safety and clinical efficacy of these drugs. Moreover, this therapy may potentially promote carcinogenesis or stimulate the growth of pre-existing undetected tumors, and this has to be checked in clinical trials [330]. Due to this, there is still a long way before activators of the PI3K/Akt pathway can be introduced into clinical practice.

Besides the activators of this pathway, substances which inhibit (e.g., LY294002) its activation are under investigation for oncology [44]. Taking into consideration all of the facts mentioned above, it seems that this drug can have a potential cardiotoxic effect. It can increase the risk of MI and post-infarct HF, but this has to be checked.

## 8. Conclusions

The PI3K/Akt pathway regulates the metabolism of cardiac cells on multiple levels, and plays an important role in the pathophysiology of myocardial infarction and the post-infarction remodeling of the left ventricle. The activation of this pathway is responsible for the cardioprotective effect of many drugs such as statins and ACEI, prescribed for patients with high cardiovascular risk or after MI. Moreover, altered activity of the PI3K/Akt pathway during diabetes may be responsible for the greater risk of death or post-MI HF development in this group of patients. The results of thebasic studies show that the modulation of this pathway by selective inhibitors or miRNA can influence both ischemic damage and post-infarction LV remodeling. Akt seems to be a key component of this signal transduction and is responsible for most of the positive effects of its activation in cardiomyocytes. There is still a long way to go before introducing drugs based on the activation or inhibition of this pathway, but this may bring significant clinical benefits.

## Data Availability

Not applicable.
